# Super-tunable, broadband up-conversion of a high-power CW laser in an engineered nonlinear crystal

**DOI:** 10.1038/s41598-017-00974-3

**Published:** 2017-04-13

**Authors:** Ameneh Bostani, Amirhossein Tehranchi, Raman Kashyap

**Affiliations:** 1 Department of Engineering Physics, Polytechnique Montreal, Montreal, QC H3T 1J4 Canada; 2 Department of Electrical Engineering, Polytechnique Montreal, Montreal, QC H3T 1J4 Canada

## Abstract

A specially-designed chirped periodically poled lithium niobate nonlinear crystal was fabricated with a phase-matching bandwidth as large as 50 nm for sum frequency generation to operate at room and higher temperatures. This device also benefits from insensitivity to laser frequency drift and fine alignment. The loosely-focused beam position of a high-power CW laser at around 1550 nm is optimized within the grating for maximum up-conversion efficiency, to realize a super-tunable source in the range of 770–778 nm by tuning a narrowband control signal over 30 nm in the communication band. This device is demonstrated to be fully phased-matched simultaneously for both second-order nonlinear up-conversion processes, namely second harmonic generation and sum frequency generation. The measurement of the generated sum-frequency power versus wavelength agrees well with the theory. The device allows for the creation of tunable broadband CW sources at shorter wavelengths with potentially high power.

## Introduction

There is increasing demand for tunable and broadband sources at shorter wavelengths due to many applications in biomedicine and spectroscopy^1–3^. Owing to the lack of suitable sources, a feasible approach is the frequency conversion of available lasers in nonlinear materials^4–6^. Recently, high-power fiber lasers have attracted huge attention^7^, however, these lasers in the form of continuous wave (CW) generally possess a bandwidth (BW) in the order of hundreds of GHz proportional to the laser output power due to the use of fiber Bragg gratings^8^ or four-wave mixing (FWM) between the different longitudinal modes^7, 9^. Further, these high-power fiber lasers are only available in limited wavelength ranges^10^. In addition, they experience a drift in their central wavelength when the output power varies^11, 12^. Nevertheless, in order to realize tunable CW sources at desired higher frequencies, up-conversion based on second-order nonlinearities using engineered quasi-phase-matching (QPM) in nonlinear crystals can be exploited with several advantages^13–17^.

There are many works on second harmonic generation (SHG) and sum frequency generation (SFG) using QPM in uniform gratings in the form of periodically poled crystals^15, 18–23^. However, uniform gratings limit the BW of up-conversion process (and consequently the tunability) as it is inversely proportional to the length of a grating^24^. Since the up-conversion efficiency (assuming an undepleted pump) is increased by the length squared, using a long grating leads to a reduction in the up-converted BW of a few-nm-wide pump and consequently results in a waste of power whilst a short grating up-converts the whole BW with a very low efficiency. Also, at higher pump powers, the use of a short grating may lead to the crystal damage in order to achieve efficiencies comparable to those of longer gratings. Moreover, as the effective period of uniform grating changes by angular rotation and temperature, fine alignment and a controlled temperature are required to obtain maximum efficiency for the central wavelength of a pump. Also, accurate temperature tuning is necessary for uniform gratings to tune the up-conversion wavelength^25, 26^. However, to convert the entire wide BW of a pump laser, especially in a tunable way, a broadband converter is required.

To increase the SHG bandwidth, some work has been done on short-range ordered ferroelectric domains^27, 28^ and modified QPM grating structures have been proposed to broaden the phase matching and multi-wavelength harmonic generation considering two domain-inverted building blocks and an aperiodic grating structure^29, 30^, although devices are not easy to fabricate^31^. The fan-out and temperature-controlled QPM devices have also been used for up-conversion bandwidth broadening by mechanical displacement and temperature control of the crystal, respectively^32, 33^ which are not appropriate for some applications^34^. Fortunately, engineered chirped and step-chirped periodically poled lithium niobate (PPLN)^35, 36^ with an SHG efficiency almost linear proportional to the grating length have been proposed to provide higher efficiency compared to very short uniform gratings to achieve the same BW. Although for the same length the conversion efficiency of a chirped grating with wide BW is less compared to that of a uniform grating with narrow BW, using a high-power source, the efficiency is not of significant concern, as power can be traded for BW. Hence, one can benefit from temperature- and fine-alignment-insensitivity of chirped grating and obtain a large conversion BW simply tolerating the drift of pump’s central-wavelength. Recently, temperature-independent broadband up-conversion (i.e., SHG and SFG) as well as cascaded with different frequency generation in step-chirped PPLN (SC-PPLN) were demonstrated for tunable monochromatic CW lasers^34, 37^ and the effect of tight focusing on broadband output spectra of such converters was reported^38, 39^.

In this paper, we demonstrate super-tunable broadband frequency up-conversion of an available high-power CW fiber laser for the first time to the best of our knowledge, in a specially-designed nonlinear crystal namely SC-PPLN device, demonstrating wideband SFG by tuning of a tunable monochromatic laser as a signal source. Our device does not need any thermal control or physical displacement and needs a simple setup for full operation. The loosely-focused beam position of the high-power CW laser is optimized within the grating for maximum up-conversion efficiency. A tunable source at shorter wavelengths is thus realized in which the central frequency is controlled by tuning the control signal laser without concern of the frequency drift of the high-power CW laser pump.

## Results

### Theory

Under the undepleted pump regime and plane wave approximation, the SFG electric field envelope in the frequency domain is given by the product of the transfer function, $\hat{D}(\omega )$ and the convolution of the pump and signal electric field envelopes^40^

1 $${A}_{3}(\omega -{\omega }_{3})=\hat{D}(\omega ).[{A}_{1}(\omega -{\omega }_{1})\ast {A}_{2}(\omega -{\omega }_{2})],$$

where *A*_*i*_ and *ω*_*i*_ are the electric field envelope in the frequency domain and angular frequency, respectively. The star sign denotes a convolution. *i* = 1, 2 and 3 denotes the pump, signal, and sum frequency (SF) waves, respectively. $\hat{D}(\omega )$ is proportional to the Fourier transform of the nonlinear modulation function, *d*(*z*) in a quasi-phase-matched structure which is engineered. In addition, the effect of focusing can be added to the transfer function integral as follows^38^

2 $$\hat{D}(\omega )=-j\gamma {\int }_{-\infty }^{+\infty }\frac{d(z)}{1+j\xi (z)}exp[j{\rm{\Delta }}k(\omega )z]dz,$$

where Δ*k* = *k*_1_ + *k*_2_ − *k*_3_ and *k*_*i*_ is the wave vector. *ξ* = 2(*z* − *f*)/*b* where *f* and *z* are the focal position and propagation axis, respectively. *b* ≅ 2*πw* ^2^ *n*_1_/*λ*_1_ is the confocal parameter where *w* and *n*_*1*_ are the beam waist, and refractive index, respectively, at the pump wavelength, *λ*_1_. $\gamma ={\omega }_{3}^{2}{\gamma }_{0}/{k}_{3}{c}^{2}$where *c* is the speed of light in vacuum and *γ*_0_ is considered to be a constant from the Boyd-Kleinman calculation for a focused Gaussian beam in a parametric interaction^41^. *d(z)* in the spatial domain can be presented as

3 $$d(z)=rect(\frac{z}{L})\times \sum _{m=-\infty }^{+\infty }(\frac{2}{m\pi }){d}_{33}\,\sin (\frac{\pi m}{2})\exp (\frac{j2\pi mz}{{\rm{\Lambda }}(z)}),$$

where *d*_*33*_ is the nonlinear coefficient of the crystal, Λ(*z*) is the period of the grating at different spatial positions, *z* and *rect* is the rectangular function.

For the input pump, we suppose a Gaussian function with a peak amplitude of *a*_*1*_ centered at *ω*_*1*_, and a BW of *σ*_*1*_ as *A*_1_ = *a*_1_ *exp*[−(*ω* − *ω*_1_)^2^/*σ*_1_]; and for the monochromic signal, we consider a Dirac delta function as *A*_2_ = *δ*(*ω* − *ω*_2_) at *ω*_2_. Therefore, the SF wave amplitude can be calculated as

4 $${A}_{3}(\omega -{\omega }_{3})=\hat{D}(\omega ){a}_{1}{\exp }[-{(\omega -({\omega }_{1}+{\omega }_{2}))}^{2}/{\sigma }_{1}],$$

which can have the same BW as the pump if the transfer function is broad enough to accommodate the Gaussian function. Therefore, with the engineering of a proper transfer function, the entire BW of a pump laser can be converted to an SF wave. However, in the case of SHG of the pump, using an auto-convolution, the spectra of *A*_2_ is replaced with *A*_1_ in Eq. (1) and the second harmonic (SH) wave amplitude can be calculated as

5 $${A}_{SH}(\omega -{\omega }_{SH})=(1/2)\hat{D}(\omega ){a}_{1}^{2}exp[-{(\omega -2{\omega }_{1})}^{2}/(\sqrt{2}{\sigma }_{1})],$$

which shows a spectral broadening of the SH amplitude in the order of the square root of two, compared to that of the input pump BW, assuming a broad enough transfer function. Thus, we expect full BW conversion of the pump in SFG and broadening of the SHG BW compared to the pump BW only if the transfer function is wide enough to accommodate both processes. The power can be also calculated from the electric field envelope in the frequency domain using $P=(\pi {w}^{2}/2nc{\varepsilon }_{0})\int {A}^{2}d\omega$ for the fundamental-harmonic (FH), SH and SF waves. Consequently, considering two Gaussian functions as inputs centered at *ω*_1_ and *ω*_2_ with the peak amplitude of *a*_1_ and *a*_*2*_, and the bandwidths of *σ*_1_ and *σ*_2_, we can calculate the power for the generated SF wave at ω_1_+ω_2_ as ${P}_{SFG}=(\pi {w}^{2}/2nc{\varepsilon }_{0}){\hat{D}}^{2}{a}_{1}^{2}{a}_{2}^{2}{\sigma }_{1}^{2}{\sigma }_{2}^{2}\pi \sqrt{\pi /2}/\sqrt{{\sigma }_{1}^{2}+{\sigma }_{2}^{2}}$.

### Design

**Figure 1:**
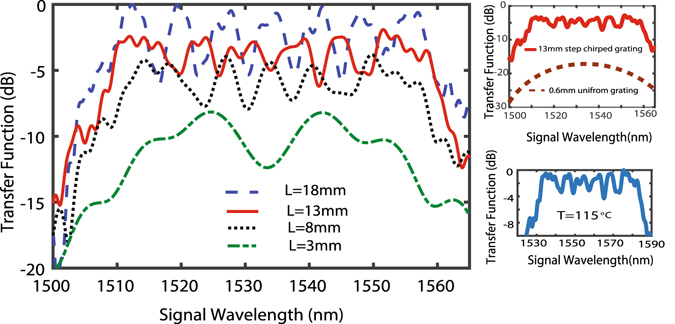
(**a**) Normalized transfer functions with bandwidths as large as 50 nm for different lengths of SC-PPLN at 25 °C, considering input pump (plane wave) at a wavelength of 1550 nm and a sweeping control signal. Longer nonlinear crystal introduces larger ripples while the up-conversion peak power increases. Therefore, there is a trade-off between the acceptable ripple height and the generated power, for the choice of length. (**b**) Equal-bandwidth transfer functions of a 0.6 mm-long uniform PPLN and a 13 mm long SC-PPLN are compared. (**c**) Transfer function for the compromised length (13 mm) at 115 °C still covers the C band.

In order to design an SC-PPLN (in congruent LN) with a wideband transfer function for SFG with a pump laser centered at 1550 nm and a swept control signal in the 30-nm C-band (1530 nm to 1560 nm), the device should work at room and possible higher temperatures to reduce possible photo-thermal damage^42^. Two pairs of minimum and maximum periods for SC-PPLNs at 25 °C and an elevated temperature, i.e., 115 °C can be calculated from the phase matching condition for SFG resulting in ${\rm{\Lambda }}=2\pi c/({\omega }_{3}{n}_{3}-{\omega }_{2}{n}_{2}-{\omega }_{1}{n}_{1})$, where *ω*_3_ = *ω*_1_ + *ω*_2_ and the refractive indices are dependent on temperature through the Sellmeier equation^43^. The minimum period at the higher temperature and the maximum period at the lower temperature are then chosen. Therefore, the SC-PPLN period change from 18.38 µm to 19.11 µm results in a 50-nm BW fully covering the C-band for temperatures from 25 °C to 115 °C. Given that our fabrication facility allows a minimum variation in the period of 100 nm (chirp step), the SC-PPLN device has to be designed in 8 sections to give an approximately linearly chirped grating. The length (*L*) of the device is then determined by a compromise between the higher efficiency and smaller ripple height. The normalized transfer function for different lengths of the SC-PPLN at room temperature is plotted in Fig. 1(a). The length of 13 mm is then selected for fabrication to reduce the ripples to ±2 dB. Longer SC-PPLNs lead to peak-to-peak ripples greater than 4 dB reaching as high as 6 dB for a length of 18 mm. Shorter SC-PPLNs reduce the efficiency without a remarkable reduction in ripples. Figure 1(b) depicts the equal-bandwidth transfer functions of a 0.6 mm-long uniform PPLN and a 13-mm-long SC-PPLN. Interestingly, the intensity for the latter is increased more than 13 dB across the entire bandwidth. Figure 1(c) shows the transfer function for the 13-mm-long SC-PPLN at 115 °C demonstrating that it tolerates a wavelength shift due to the temperature change but still covers the C-band.

Our device works like a 2D nonlinear photonic crystal^13, 14, 44^ as it has a large acceptance angle. Briefly, a uniform nonlinear crystal in 1D can have a very large bandwidth if it is very short, behaving similar to a photonic crystal in 1D as it has a small angular acceptance angle, but with a poor efficiency due to its limited length. Other structures, such as with a radially periodic nonlinearity can behave as “nonlinear photonic crystal” in 2D, as they have a nonlinear response potentially over 360 degrees, however, these structures do not necessarily have large bandwidths^44^. Our device behaves differently in that being chirped, has a large bandwidth and a much larger acceptance angle than the uniform grating would have for a single section of the SC-PPLN, as well as better theoretical efficiency. The device tolerates ±11.4° yaw to obtain a half BW, which is almost 2 times greater than is possible for a short uniform PPLN with a single section length in addition to benefiting from a higher efficiency as large as 5 dB.

**Figure 2:**
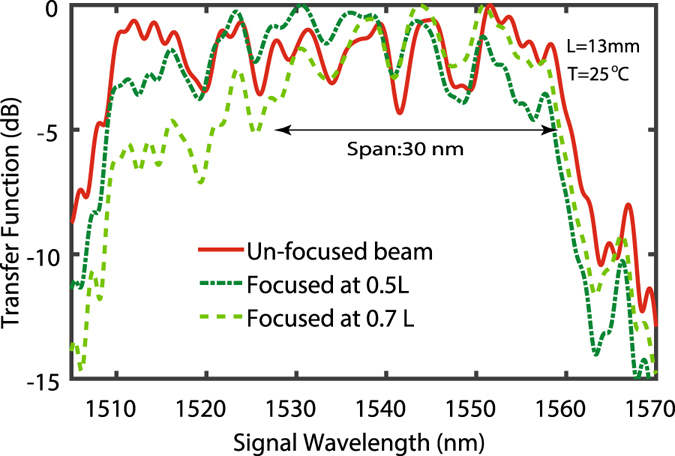
Normalized transfer functions of SC-PPLN using plane wave (solid line) and focused beams at two different positions of 0.5 *L* and 0.7 *L* along the grating. Focusing suppresses the side-lobes in the transfer function far from the phase-matching wavelength of the grating in the focusing point. The focal point is optimized to be at 0.7 *L* to achieve the maximum up-conversion power for 1550 nm, and to maintain the required 30 nm working bandwidth.

In addition, we can simulate the role of focusing on the transfer function, to approach the real experimental conditions. The effect of different degrees of focusing and the position of the focus within the grating were already examined in ref. 30. Focusing can suppress the side-lobes in the transfer function of the SC-PPLN far from the phase-matching wavelength of the grating in the focusing point. In Fig. 2, the transfer functions for a loosely focused light beam with a waist of 70 µm located at 0.5 × *L* and 0.7 × *L* are plotted for a 13-mm-long SC-PPLN at room temperature, using a fixed pump at 1550 nm and a swept signal. For comparison, the transfer function of the ideal plane wave is also shown in Fig. 2. In the case of focused beams, the focal point is optimized to be at 0.7 × *L* within the grating to achieve the maximum up-conversion power for 1550 nm, and to maintain the required 30-nm working BW. The transfer function is suppressed in the range of 1510 nm to 1530 nm, but this is not a concern as it is outside the designed working range.

### Experimental Results

**Figure 3:**
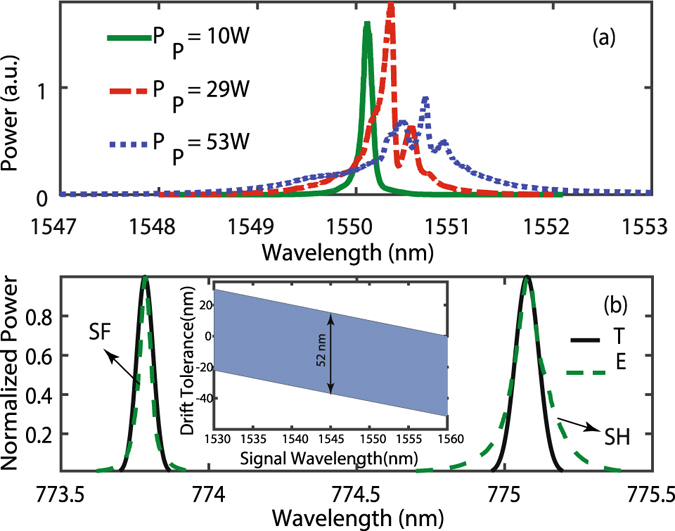
(**a**) Pump spectra for different integrated pump powers. (**b**) Theoretical (T) and experimental (E) results of simultaneous SFG and SHG when the pump and control signal are at 1550.16 nm and 1545 nm, respectively and the pump power is around 20 W. The inset shows the region of tolerable drift in the pump central wavelength at 1550 nm for different signal wavelengths. For the pump central wavelength at 1550 nm, the drift bandwidth is 52 nm for the signal within the C-band.

The spectra of the CW pump laser at three output powers are shown in Fig. 3(a). The FWHM of the laser increases from ~0.1 nm at 10 W to ~1 nm at 53 W due to the FWM within the fiber laser. In addition, it shows a spectral-shift (with a peak wavelength drift <0.75 nm) toward longer wavelengths as apparent in Fig. 3(a). The experimental spectra of simultaneous SFG and SHG for the high-power laser centered around 1550 nm and the signal at 1545 nm are shown in Fig. 3(b).

The results are compared with the computed spectra for SFG and SHG simulated by the real pump spectrum and a monochromatic wavelength at 1545 nm as the inputs to the SC-PPLN. The SF wave, centered around 773.78 nm, has a spectrum corresponding to the pump BW as expected from the theory. The SH BW centered at 775.08 is ~1.4 times broader compared to the SF as it is proportional to the auto-convolution of the FH beam in the agreement with the presented theory. Also, both *χ* ^(2)^ processes tolerate the drift easily in the central wavelength of the pump. Based on the up-conversion simulation, the pump central wavelength (at 1550 nm) can undergo −37 nm to + 15 nm drift for the signal at a wavelength of 1545 nm as shown in the inset of Fig. 3(b).

**Figure 4:**
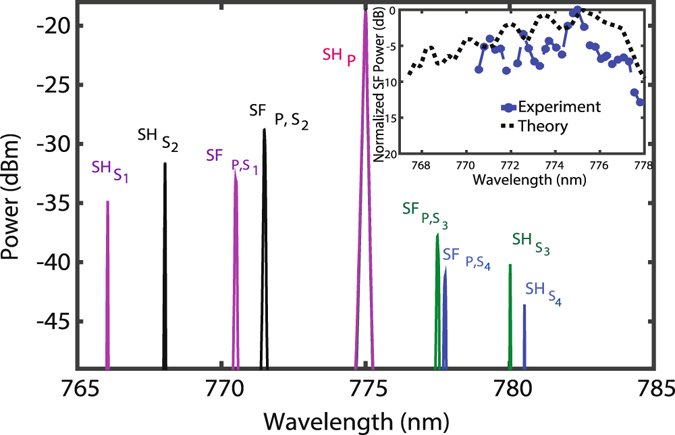
Overlaying of four experimentally observed spectra including SH of a pump (SHP) at *λ*_1_ = 1550 nm and SH of four tuned signals at *λ*_S1_ = 1532 nm, *λ*_S2_ = 1536 nm, *λ*_S3_ = 1560 nm and *λ*_S4_ = 1561 nm named as SH_S1_, SH_S2_, SH_S3_ and SH_S4_ and SF of the pump and signals presented by SF_P,S1_, SF_P,S2_, SF_P,S3_ and SF_P,S4_. The inset shows theoretical and experimental results for normalized SF power versus wavelength demonstrating the tunability of the device over >8 nm.

Next, the control signal is tuned when the pump wavelength is fixed at 1550 nm and simultaneous SH spectra of the pump and signal and associated SF spectrum are observed. In Fig. 4, four overlaying spectra of SH and SF for four different control signals centered at 1532 nm, 1536 nm, 1560 nm, and 1561 nm are shown. Each SF wave has a broader and narrower BW than that of the corresponding signal SH wave and pump SH wave, respectively, as predicted by Eqs (4) and (5). Filtering is necessary to have a single peak emission for SFG. The maximum power of SF waves alters for different signal wavelengths as expected from the ripply transfer function of the SC-PPLN, shown in Fig. (2).

The simulated and experimental results (at 25 °C) for normalized maximum SF power versus wavelength is plotted in the inset of Fig. 4 for a pump at 1550 nm and a swept control signal. The measured SF central wavelength is successively tuned from ~770 nm to 778 nm by tuning the control signal from 1530 nm to 1560 nm, confirming an experimentally tunable BW of ~8 nm for the SF whilst for an equivalent-length, uniform PPLN, it is only 0.41 nm, demonstrating the super-tunability of our SC-PPLN. However, the difference between experimental and simulated results comes from accumulated phase differences in the fabrication and errors involved in the poling process. The simulated response in the inset is shown in a wider range than the experimental one as the SC-PPLN is designed to work also at higher temperatures. Theoretical calculation for the SF power using Eq. 1 and assuming Gaussian functions (approximately) with the integrated powers and bandwidths of (*P*_1_, *σ*_1_) and (*P*_2_, *σ*_2_) for the pump and signal, respectively leads to ${P}_{SF}\propto {P}_{1}{P}_{2}{\sigma }_{1}{\sigma }_{2}/{({\sigma }_{1}^{2}+{\sigma }_{2}^{2})}^{1/2}$ which results in a small SF power where *σ*_1 _≫ *σ*_2_ even if *P*_1_ is comparable to *P*_2_. For instance, considering a typical 1-W signal power at 1542 nm and a 3-W integrated power of the pump, the maximum integrated power in the generated SF power at 773.2 nm is calculated and measured to be around 75 µW resulting in an SFG efficiency of ~0.025/kW.

**Figure 5:**
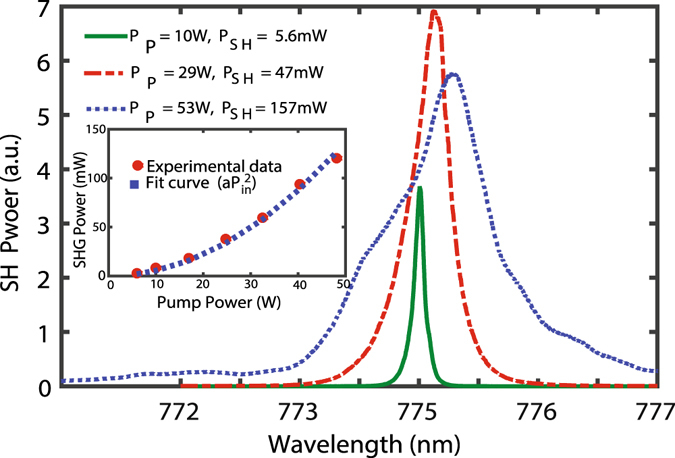
SH spectra for 3 pump powers shown in Fig. 3(a). The inset shows output SH power in mW versus input FH pump power in W. Quadratic SH power with respect to the pump power is realized and the *a* coefficient is ~0.056/kW.

In a special case, by turning the control signal off, an SH proportional to the auto-convolution of the high-power pump is generated. The SH spectra for three pump powers (already illustrated in Fig. 3a) are shown in Fig. 5. It is obvious that the SC-PPLN performs well for full-BW frequency-doubling of the FH wave, and is also able to handle the pump spectral-shift. The generated SH power is measured and plotted versus the input power in the inset of Fig. 5.

The SH power changes quadratically with the FH power as is expected from the un-depleted pump theory. The quadratic fit curve to the experimental data is shown in the inset and the estimated quadratic coefficient, *a* is 0.056/kW, which is comparable with the theoretical calculation of the efficiency using the following equation $a={P}_{SH}/{P}_{P}^{2}\,=\,8\pi /{\lambda }_{1}^{2}c{\varepsilon }_{0}{n}_{SH}{n}_{1}^{2}{w}^{2}{[{\int }_{0}^{L}d(z){e}^{j\Delta kz}dz]}^{2}\,$for an SC-PPLN, where *d*_33_ is 21 pm/V, the free space permittivity, *ε*_0_ is 8.85 × 10^−12^ F/m, *c* is 3 × 10^8^ m/s, *w* is 70 μm, and ${n}_{SH}$ is the refractive index of LN at the SH wavelength, respectively. The calculation gives the coefficient, *a* in the range of 0.048/kW to 0.12/kW which depends on the chirp step, SC-PPLN length, working wavelength and temperature. No photorefractive damage was seen at 25 °C for different FH powers, however, we increased the temperature from room temperature to 115 °C. The change in the SH power is less than 3 dB over a 90 °C temperature-tuning BW in a fixed-position alignment and thus, the device does not require a temperature controller. It is worth noting that the similar BW for an equivalent-length uniform PPLN is calculated to be only around 4 °C.

## Discussion

In this paper, it was demonstrated both experimentally and theoretically that by engineering the chirped grating in a nonlinear crystal and designing the transfer function larger than the signal tuning span, all spectral components of a few-nm-wide pump and a tunable CW signal can be phase-matched over the transfer function’s bandwidth to generate an SF wave with a corresponding bandwidth as the pump wave. For the SC-PPLN, a 50 nm wide transfer function was obtained to cover the C-band at room temperature and higher temperatures (up to 115 °C). In this device, SFG phase-matching of the control signal swept over 30-nm in the C-band with a high-power CW pump at 1550 nm resulted in the generation of a super-tunable SF wave over ~8 nm around 775 nm. Also, the loosely-focused beam position along the grating is moved to maximize the up-conversion efficiency around 1550 nm in the transfer function. The focusing position has been found to be optimized at 0.7 × *L* of the grating. The measured generated SF power versus wavelength is in a good agreement with the theory. Although the SF response has ripple due to the transfer function profile, apodization of the SC-PPLN grating can remove the ripples^35, 45^. In a special case of turning the signal off and obtaining frequency doubling of the pump alone, the SH wave with a broader bandwidth than that of the pump was achieved which is the consequence of the auto-convolution of the pump wave distribution function in the frequency domain^40^. Quadratic SH power with respect to the pump power was realized and the quadratic coefficient was measured to be ~0.056/kW. The device is insensitive to pump-spectrum drift and does not need a temperature controller and also can tolerate ± 11.4° yaw, i.e. an input acceptance angle. The current engineering technique can also be applied to other wavelength ranges and materials as long as the phase-matching conditions are met.

## Method

**Figure 6:**
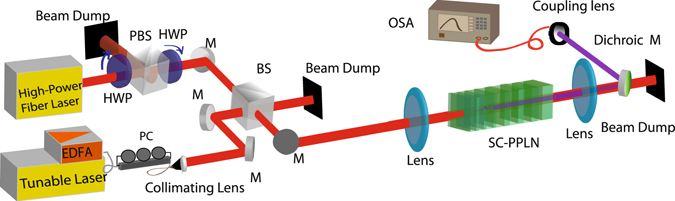
Experimental setup to evaluate SFG in an SC-PPLN with a high-power fiber laser as a pump and a tunable CW laser as a control signal, EDFA: erbium doped fiber amplifier, PC: polarization controller, M: mirror, BS: beam splitter, PBS: polarization beam splitter, HWP: half-wave plate and OSA: optical spectrum analyzer. The light from the high-power laser is combined with the light from the tunable laser after polarization controlling, in a 50:50 beam splitter and loosely focused in the SC-PPLN. The higher harmonics are separated by a dichroic mirror and coupled to an optical spectrum analyzer. SHG in the SC-PPLN is evaluated using the high-power laser by turning the tunable laser off.

The fabricated mask based on our design is used in the photolithography on a + *z* face of an undoped, 0.5-mm-thick, *z*-cut, optical grade LN crystal. Electric-field poling at room temperature, a common technique for the fabrication of QPM devices, especially for LN is used^37, 46^. A dc electric field (22 kV/mm) higher than the coercive field strength of LN in the shape of pulses with duration of 0.5 ms was applied to the patterened LN. The fabricated SC-PPLN then was cut, polished and placed in the experimental setup as shown in Fig. 6.

A CW erbium fiber laser (ELR-75-1550) from IPG Photonics with a central emission wavelength around 1550 nm with multiple longitudinal modes having random phases is used as a high-power FH pump. A combination of a half-wave plate and a polarization beam splitter (PBS) cube in front of the pump beam is placed to adjust the power. After the PBS, another half-wave-plate is located to fix the light polarization before entering into the SC-PPLN.

The pump beam is combined in a 50:50 beam splitter with the control signal coming via a fiber-based polarization controller from another device: a JDSU tunable laser amplified by a C-band EDFA. The signal beam from the fiber, collimated by a spherical lens enters the beam splitter cube. The two combined beams (the pump and signal) are loosely focused into the SC-PPLN by a lens with a focal length of 12.5 cm (at around 1550 nm). The output SF and SH waves were separated from the pump by a dichroic mirror, then collimated and coupled into the output fiber and analyzed using an optical spectrum analyzer and a power meter.
